# A randomised, double-blind, placebo-controlled trial of tropisetron in patients with schizophrenia

**DOI:** 10.1186/1744-859X-9-27

**Published:** 2010-06-24

**Authors:** Akihiro Shiina, Yukihiko Shirayama, Tomihisa Niitsu, Tasuku Hashimoto, Taisuke Yoshida, Tadashi Hasegawa, Tadashi Haraguchi, Nobuhisa Kanahara, Tetsuya Shiraishi, Mihisa Fujisaki, Goro Fukami, Michiko Nakazato, Masaomi Iyo, Kenji Hashimoto

**Affiliations:** 1 Department of Psychiatry, Chiba University Hospital, Chiba, Japan; 2 Department of Mental Health, Teikyo University Chiba Medical Center, Chiba, Japan; 3 Department of Psychiatry, Chiba University Graduate School of Medicine, Chiba, Japan; 4 Division of Clinical Neuroscience, Chiba University Center for Forensic Mental Health, Chiba, Japan

## Abstract

**Background:**

Cognitive deficits in schizophrenia are associated with psychosocial deficits that are primarily responsible for the poor long-term outcome of this disease. Auditory sensory gating P50 deficits are correlated with neuropsychological deficits in attention, one of the principal cognitive disturbances in schizophrenia. Our studies suggest that the α7 nicotinic acetylcholine receptor (α7 nAChR) agonist tropisetron might be a potential therapeutic drug for cognitive deficits in schizophrenia. Therefore, it is of particular interest to investigate the effects of tropisetron on the cognitive deficits in patients with schizophrenia.

**Methods:**

A randomised, placebo-controlled trial of tropisetron in patients with schizophrenia was performed. A total of 40 patients with chronic schizophrenia who had taken risperidone (2 to 6 mg/day) were enrolled. Subjects were randomly assigned to a fixed titration of tropisetron (n = 20, 10 mg/day) or placebo (n = 20) in an 8-week double-blind trial. Auditory sensory gating P50 deficits and Quality of Life Scale (QLS), Cambridge Neuropsychological Test Automated Battery (CANTAB), and Positive and Negative Syndrome Scale (PANSS) scores were measured.

**Results:**

In all, 33 patients completed the trial. Tropisetron was well tolerated. Administration of tropisetron, but not placebo, significantly improved auditory sensory gating P50 deficits in non-smoking patients with schizophrenia. The score on the rapid visual information processing (sustained visual attention) task of CANTAB was significantly improved by tropisetron treatment. Total and subscale scores of PANSS were not changed by this trial. QLS scores in the all patients, but not non-smoking patients, were significantly improved by tropisetron trial.

**Conclusions:**

This first randomised, double-blind, placebo-controlled trial supports the safety and efficacy of adjunctive tropisetron for treatment of cognitive deficits in schizophrenia.

## Background

Cognitive deficits in schizophrenia are frequently severe and strongly correlated with decreased functional outcome and quality of life (QOL) [1-5]. Atypical antipsychotics have been shown to provide an improvement in several domains of cognitive function, especially working memory, executive function, and attention [2,6]. However, many patients, even if they are medicated with atypical antipsychotics, fail to recover from cognitive deficits, resulting in a failure of their reintegration into society.

Several lines of evidence suggest that the α7 subtype of the nicotinic acetylcholine receptors (α7 nAChRs) plays an important role in the mechanism of auditory P50 gating deficits of schizophrenia, and that α7 nAChR agonists are potential therapeutic drugs for the deficient inhibitory processing of the P50 auditory evoked potential that contributes to the cognitive deficits in schizophrenia [7-17]. Tropisetron (Nabovan), a potent serotonin-3 (5-hydroxytryptamine; 5-HT_3_) receptor antagonist, is widely used in the treatment of patients with chemotherapy-induced or postoperative nausea and vomiting [18]. It has also been reported that tropisetron is a partial agonist at α7 nAChRs with a high affinity (Ki = 6.9 nM for α7 nAChRs; Ki = 5.3 nM for 5-HT_3 _receptors) [19]. We previously reported that tropisetron normalises deficient auditory gating in DBA/2 mice and that this effect of tropisetron was blocked by coadministration of the selective α7 nAChR antagonist methyllycaconitine (MLA) [20]. Furthermore, we reported that tropisetron or the selective α7 nAChR agonist SSR180711, but not the selective 5-HT_3 _receptor antagonist ondansetron, could ameliorate phencyclidine (PCP)-induced cognitive deficits in mice, and that the effects of tropisetron or SSR180711 were blocked by coadministration of MLA [21,22]. In addition, we reported that a single oral administration of tropisetron (10 mg) could improve deficits in auditory P50 suppression in Japanese schizophrenic patients [23]. The neuropharmacological and neurobiological effects of tropisetron demonstrated by our group suggest the need for future studies to assess the effects of tropisetron on cognitive dysfunction in schizophrenia, because P50 deficits have been previously associated with attentional deficits in schizophrenia [24]. These attentional deficits are in turn associated with psychosocial deficits that are primarily responsible the poor long-term outcome of schizophrenia [1].

This randomised, double-blind, placebo-controlled study was undertaken to examine whether adjunctive tropisetron could improve cognitive deficits and other clinical variables in patients with schizophrenia. A dose of 10 mg tropisetron was chosen since this dose has been proven effective in deficits in auditory P50 suppression of schizophrenia [23]. Risperidone was used as an antipsychotic drug because risperidone does not exhibit significant α7 nAChR agonism or 5-HT_3 _receptor antagonism.

## Methods

### Participants

The subjects were 40 patients (19 males and 21 females; age: 35.1 ± 7.63 years (mean ± SD); age range: 21 to 48 years) with schizophrenia meeting the *Diagnostic and Statistical Manual of Mental Disorders, fourth edition (text revision) *(DSM-IV TR) criteria [25] who were outpatients of Chiba University Hospital, Chiba, Japan (Table 1). As required by the selection criteria, they were clinically stable outpatients with no medical or neurological illness, or alcohol or other substance dependence. To be eligible for the trial, all patients had to have been taking the atypical antipsychotic drug risperidone (2 to 6 mg/day) for at least 8 weeks, and the dose of this antipsychotic was not changed during the trial. Concomitant psychiatric medications were permissible (antidepressants, mood stabilisers, anticholinergics, and others) (Table 2), provided that patients were receiving stable doses of all of these medications in the 4 weeks preceding the trial and throughout the entire duration of the study. In all, 10 patients in the placebo group and 11 patients in the tropisetron group had taken anticholinergics (biperiden or trihexyphenidyl hydrochloride) during the trial. The difference between the two groups in mean dosage of anticholinergics was not statistically significant (unpaired Student t test; *P *= 0.51). Furthermore, 8 patients in the placebo group and 12 participants in the tropisetron group had taken benzodiazepines during the trial. However, five patients in the placebo group and three patients in the tropisetron group had taken benzodiazepines during the day. Moreover, one patient had taken lithium, and another patient had taken milnacipran and trazodone in the placebo group. One patient had taken valproic acid, and another patient had taken carbamazepine in the tropisetron group. None of the patients had taken tricyclic antidepressants. None of the patients (except dropouts) altered his or her medications during the trial. For polypharmacy, four patients in the placebo group and six patients in the tropisetron group had taken two or more kinds of drugs within anticholinergics, benzodiazepine during the day, antidepressants, or mood stabilisers. Any change in psychiatric medications at any point during the study rendered a patient ineligible to continue participation.

**Table 1:** Characteristics of subjects

	Placebo group	Tropisetron group	*P *value
Sex (M/F)	10/10	9/11	NS^a^
Age	35.15 ± 8.54	34.96 ± 6.82	NS^b^
Subtype	Disorganised 1	Disorganised 0	NS^a^
	Catatonia 1	Catatonia 0	
	Paranoid 13	Paranoid 11	
	Undifferentiated 2	Undifferentiated 3	
	Residual 3	Residual 6	
Duration of illness	9.79 ± 6.43	12 ± 8.67	NS^b^
Dose of risperidone	3.8 ± 1.58	4.03 ± 1.59	NS^b^
No. smoking	5 (25%)	6 (30%)	NS^a^
Full IQ	87.00 ± 16.80	87.68 ± 18.86	NS^b^
PANSS score	Positive: 11.8 ± 3.12	Positive: 11.85 ± 3.22	NS^b^
	Negative: 18.45 ± 6.66	Negative: 18.95 ± 5.23	
	General: 34.3 ± 7.53	General: 33.75 ± 8.69	
	Total: 64.55 ± 15.17	Total: 64.55 ± 15.65	
QLS total score	77.05 ± 15.8	72.2 ± 13.95	NS^b^
Rate of completion	17 (85%)	16 (80%)	NS^a^
Reason for dropout	Physical illness: 1	Non-adherence: 2	
	Worsening of illness: 1	Unknown: 1	
	Adverse effects: 1 (extrapyramidal signs)	Adverse effect: 1 (chest pain)	

Data show the mean ± SD.

^a^χ^2 ^test; ^b^Student t test.

NS = not significant; PANSS = Positive and Negative Syndrome Scale; QLS = Quality of Life Scale.

**Table 2:** Other medications used by patients

Drugs	Placebo group (N = 20)	Tropisetron group (N = 20)
Anticholinergics	N = 10, (mean 2.2 mg/day)	N = 11, (mean 1.9 mg/day)
Benzodiazepines	N = 8, (with 5 during the day)	N = 12, (with 3 during the day)
Other medications	Lithium (N = 1), milnacipran and trazodone (N = 1)	Valproic acid (N = 1), carbamazepine (N = 1)

### Ethics

The trial was approved by the Institutional Review Board of Chiba University Hospital (Chiba, Japan), and registered on the Clinical Trials Registry of the University hospital Medical Information Network (UMIN, Tokyo, Japan). All subjects provided written informed consent for their participation in the study after the procedure had been fully explained to them.

### Study design

This double-blind, placebo-controlled trial used a randomisation procedure established by UMIN (UMIN 000003084). A total of 20 subjects received 10 mg/day tropisetron (Nabovan; Novartis Pharma KK, Tokyo, Japan), and 20 subjects received a matching placebo capsule. Medications were dispensed in blister packs by the Department of Pharmacy of Chiba University Hospital.

### Clinical variables and cognition

The Positive and Negative Syndrome Scale (PANSS) was used to quantify the severity of psychotic symptoms [26]. Drug-induced extrapyramidal symptoms were assessed using the Drug-Induced Extrapyramidal Symptoms Scale (DIEPSS) [27]. Patients were closely monitored for any adverse events or clinical deterioration. Quality of Life Scale (QLS) [28] scores were also measured. PANSS, DIEPSS and QLS scores were measured twice (at baseline and 8 weeks).

Cognitive function in patients with schizophrenia was measured by the Cambridge Neuropsychological Test Automated Battery (CANTAB), which consists of a series of interrelated computerised non-verbal tests of memory, attention, and executive function [29-31]. The CANTAB tests used in this study were the pattern recognition memory (PRM: recognition memory for patterns), spatial recognition memory (SRM: recognition memory for spatial locations), delayed matching to sample (DMS: simultaneous and delayed perceptual matching), span length of spatial span (SSP: working memory capacity), spatial working memory (SWM: working visuospatial memory and strategy use), stocking of Cambridge (SOC: spatial planning and motor control), intra-extra dimensional set shifting (IED: rule acquisition and attentional set shifting), and rapid visual information processing (RVP; sustained visual attention) [31] tests. In this study, measurement by CANTAB was performed at baseline and 8 weeks.

### Auditory sensory gating P50

Subjects were seated in a comfortable recliner and instructed to relax with their eyes open and to focus on a fixation point. The testing took place in a quiet, lighted room. The subject was monitored visually and by electroencephalogram (EEG) for signs of sleep or slow wave activity, which prompted the experimenter to speak briefly with the subject. The 120 pairs of auditory clicks were presented at a rate of 1 pair every 10 s, with a 500-ms interclick interval. The stimuli were 1 ms square waves amplified to 70 dB SPL. Recordings were performed with gold disc electrodes at Fz, Cz, and Pz with a forehead ground and linked earlobe reference. Data from the Cz site are presented, since recordings at Cz best discriminate schizophrenia patients from normal subjects [32]. Eye movements and blinks were monitored by electro-oculographic (EOG) recording. The resistance of all electrodes was less than 10 kohm. EEG activity was recorded by an MEB2208 8-channel system (Nihon Kohden, Tokyo, Japan) with filters at 0.5 and 100 Hz. Data were acquired at a 500 Hz digitisation rate, and individual trials were stored to disk for analysis. Individual trials were rejected if the EEG or EOG voltage was greater than ±70 μV, which is generally indicative of excessive muscle activity, eye movements, or other artefacts. The conditioning P50 wave was identified by a rater blind to the subject identity and the treatment conditions as the most positive peak between 40 and 90 ms after the conditioning stimulus. The test P50 wave was identified as the positive peak after the test stimulus that was closest in latency to the conditioning P50. The amplitude was defined as the difference between the positive peak and the preceding negative trough for both waves. The P50 T/C ratio was calculated by dividing the test P50 amplitude by the conditioning P50 amplitude. Auditory sensory gating P50 was measured twice in all patients (at baseline and 8 weeks). Due to EEG noise, we could not measure the P50 in three patients of the placebo group.

### Statistical analysis

The data were expressed as the mean ± SD. Data analysis was performed using PASW Statistics 18.0 (formerly SPSS statistics; SPSS, Tokyo, Japan). The differences between groups were evaluated by χ^2 ^test and Student t test. The data on P50 and QLS in each group were assessed by paired Student t test. Values of *P *< 0.05 were considered to indicate statistical significance in these analyses. First, the data on the eight domains of CANTAB were assessed by repeated two-way analysis of variance (ANOVA). Next, the data of CANTAB in each group were assessed by paired Student t test since there was no significant effect. We used a Bonferroni correction for multiple comparisons of CANTAB data.

### Trial registration

University hospital Medical Information Network (UMIN) Clinical Trials registry No.: UMIN 000003084 http://www.umin.ac.jp.

## Results

### Participants

Table 1 shows the characteristics of all participants. There were no differences on their distribution in sex, age, diagnosis subtype, duration of illness, dosage of risperidone, full IQ, severity of symptoms measured with PANSS, or QLS total score (Table 1). In all, 33 participants completed the trial. Four patients in the tropisetron group dropped out due to non-adherence (n = 2) and adverse effects (chest pain n = 1, unknown n = 1); and three patients in the placebo group dropped out due to physical illness (influenza n = 1), worsening of illness (n = 1), and adverse effects (extrapyramidal side effects n = 1) (Table 1).

### Adverse events

There were no significant effects of drug treatment on vital signs, ECG, or the results of haematology measurements or serum chemistry tests. One patient in the tropisetron group complained of moderate constipation and needed to take a laxative. One female patient in the tropisetron group complained of mild chest pain, although no changes were observed in her ECG (Table 1). After conferring with the investigator she decided to discontinue the trial. Shortly after discontinuation her symptoms vanished without any medical intervention or worsening of her mental or physical status. None of the patients showed any remarkable change in their DIEPSS severity score throughout the trial, and no other adverse events were observed. Tropisetron was thus well tolerated in this trial.

### Auditory sensory gating P50 deficits

**Figure 1:**
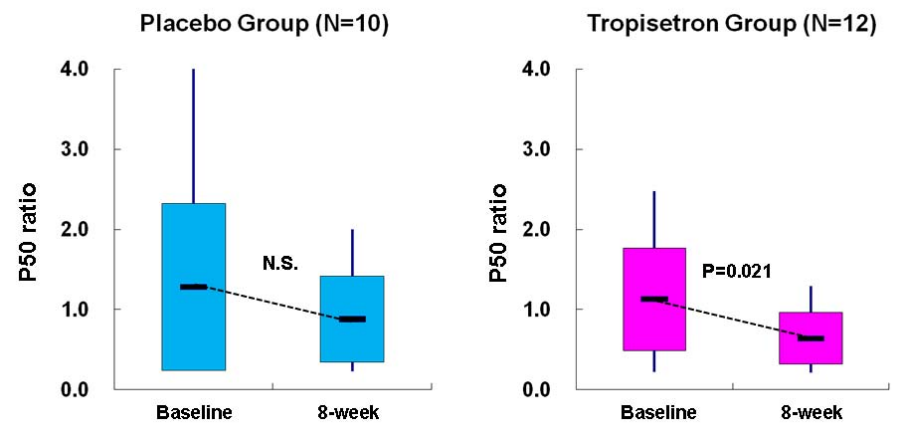
**Effect of tropisetron on auditory sensory gating P50 deficits in non-smoking patients with schizophrenia**. The ratio of test P50 amplitude to conditioning amplitude was measured at baseline and 8 weeks after placebo or tropisetron treatment. Tropisetron (t = 2.70, degrees of freedom (df) = 11, *P *= 0.021), but not placebo (t = 1.66, df = 9, *P *= 0.132), significantly decreased the P50 ratio in non-smoking patients with schizophrenia. Data are from the placebo group (n = 10) and the tropisetron group (n = 12).

Administration of tropisetron (n = 16, 10 mg/day for 8 weeks; t = 3.24, degrees of freedom (df) = 15, *P *= 0.006), but not placebo (n = 14, t = 0.570, df = 13, *P *= 0.578), significantly improved auditory sensory gating P50 deficits in patients with schizophrenia (Additional file 1). Next we analysed the data for non-smokers, since it is well known that smoking can affect the P50 suppression ratio and cognition in schizophrenia [33-35]. Administration of tropisetron (n = 12, 10 mg/day for 8 weeks; t = 2.70, df = 11, *P *= 0.021), but not placebo (n = 10, t = 1.66, df = 9, *P *= 0.132), significantly improved auditory sensory gating P50 deficits in non-smoking patients with schizophrenia (Figure 1). Subsequently, we analysed the data of non-smoking patients with schizophrenia.

### Cognitive deficits

**Figure 2:**
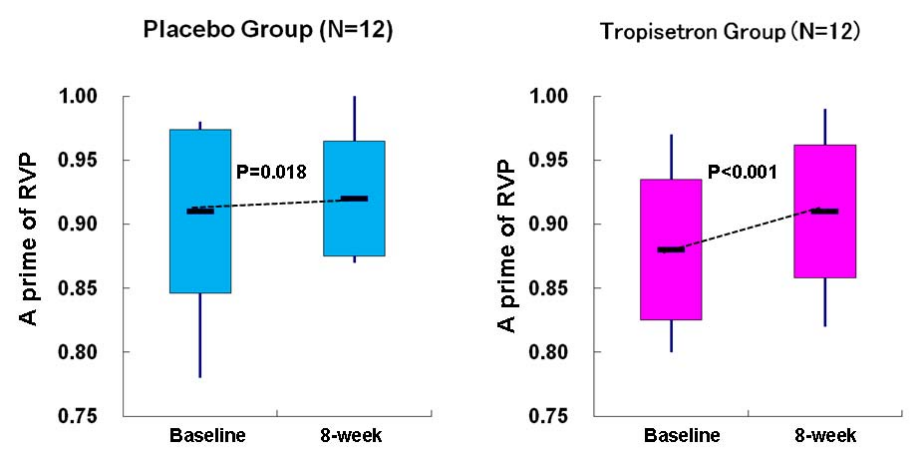
**Rapid visual information processing (RVP) scores on Cambridge Neuropsychological Test Automated Battery (CANTAB) subtests for non-smoking patients with schizophrenia during treatment placebo and tropisetron**. There were significantly different scores for RVP (t = -5.78, degrees of freedom (df) = 11, *P *< 0.001) in the tropisetron group, but not the placebo group. Data are from the placebo group (n = 12) and the tropisetron group (n = 12).

Repeated two-way ANOVA showed that performance on the eight domains of the CANTAB did not differ between tropisetron group and placebo group. Therefore we performed a secondary analysis using a paired Student t test in each group of non-smoking patients. Some subtests of CANTAB were significantly improved after the treatment. In the non-smoking placebo group (n = 12), the scores of correct rate in all trials of DMS (t = -3.94, df = 11, *P *= 0.002), SSP (t = -2.60, df = 11, *P *= 0.025), and a prime of RVP (t = -2.77, df = 11, *P *= 0.018) were improved after placebo treatment, but SSP and RVP were not significant after a Bonferroni correction. However, DMS was still significant after a Bonferroni correction, suggesting that a practice effect was operative. Furthermore, the scores for RVP were significantly improved after treatment with tropisetron (t = -5.78, df = 11, *P *< 0.001); which was still significant after a Bonferroni correction (Figure 2). Other subtests of CANTAB were not changed by this trial. In addition, we did not observe any significant correlation between P50 changes and RVP changes in the non-smoking patients with schizophrenia.

### Psychotic symptoms and QLS

Total and subscale scores (positive symptoms, negative symptoms, or general psychopathological symptoms) of PANSS were not altered by treatment with tropisetron or placebo (Additional file 2). Furthermore, administration of tropisetron significantly (t = -2.42, df = 15, *P *= 0.029) increased QLS total scores in total patients (n = 16) with schizophrenia, whereas administration of placebo did not change QLS total scores (t = -0.72, df = 13, *P *= 0.487) (Additional file 3). However, administration of tropisetron did not alter QLS total scores in non-smoking patients (n = 12). In addition, we did not observe any significant correlation between P50 changes and QLS score changes in the total or non-smoking patients with schizophrenia.

### Effects of tropisetron on smoking patients

Moreover, we did not observe any effect of tropisetron on the P50 ratio, psychotic symptoms, QLS, or cognitive functions in the smoking patients (tropisetron group n = 4; placebo group n = 5), although the sample number in each group was too small to reach any definitive conclusions in regard to these parameters. In addition, tropisetron did not affect smoking status (for example, the number of cigarettes) in the smoking patients (n = 4).

## Discussion

The major findings of this double-blind, placebo-controlled study are that administration of tropisetron, but not placebo, significantly improved auditory sensory gating P50 deficits in patients with schizophrenia, and that tropisetron had a significant impact on the sustained visual attention measured with the RVP subtest of CANTAB in non-smoking patients. Previously, we reported that the effect of a single administration of tropisetron (10 mg) on P50 deficits in patients with schizophrenia was significant only for the non-smokers [23], indicating that smoking status can affect P50 deficits in schizophrenia. In the present study, we also found that administration of tropisetron (10 mg/day for 8 weeks), but not placebo, significantly improved P50 deficits in non-smoking patients with schizophrenia, suggesting that chronic as well as acute administration of tropisetron (10 mg) can improve P50 deficits in non-smoking patients with schizophrenia. Interestingly, we found that sustained visual attention as measured with the RVP subtest of CANTAB in non-smoking patients was significantly improved by tropisetron treatment, but not by placebo treatment. It has been reported that the α7 nAChR agonist 3-(2,4-dimethoxybenzylidene) anabaseine (DMXB-A) had effects on auditory P50 deficits, attention/vigilance and working memory Measurement and Treatment Research to Improve Cognition in Schizophrenia (MATRICS) [36,37] domains in stable and non-smoking patients with schizophrenia [13,14]. Auditory sensory P50 deficits have also been shown to be associated with attentional deficits in schizophrenia [24]. Unexpectedly, we did not observe any significant correlation between P50 changes and RVP subtest changes in the non-smoking patients because of the small sample size. A further study using a large sample size will be necessary. Taken together, it is likely that the improvement of P50 deficits by tropisetron is involved in the beneficial effects of this drug on attention. Furthermore, the results of this trial suggest that stimulation at α7 nAChRs by α7 nAChR agonists can lead to improvement in auditory P50 deficits and aspects of cognitive performance such as attention.

Several studies strongly suggest that cognitive deficits have a major impact on QOL in patients with schizophrenia [1,4,38,39]. A recent review of longitudinal studies demonstrated that cognition deficits are associated with functional outcomes in schizophrenia, and that cognitive assessment predicts later functional outcomes in patients, suggesting a rationale for psychopharmacological interventions for cognitive deficits [4]. It is thus significant that, in the present study, an 8-week treatment with tropisetron could improve the QOL in total patients with schizophrenia although this is not significant in the non-smoking patients. In addition, we did not observe any significant correlation between P50 changes and QLS score change in the non-smoking patients because of small sample size. A further study using a large sample size will be necessary. Freedman *et al. *[14] reported that treatment with DMXB-A significantly improved two subscales (for example, alogia and anhedonia) of the Scale for the Assessment of Negative Symptoms (SANS), but not the total score of the Brief Psychiatric Rating Scale (BPRS). The effects of DMXB-A on negative symptoms are also noteworthy, as these negative symptoms are generally resistant to antipsychotic drugs. However, in this trial, we did not observe any effect of tropisetron on the positive symptoms or negative symptoms scores of PANSS. Further detailed studies regarding the effects of tropisetron on psychotic symptoms such as negative symptoms will therefore be necessary.

Tropisetron is also a potent antagonist at 5-HT_3 _receptors. At present, it is unclear whether improvement of P50 deficits by tropisetron is mediated via direct agonist effects on α7 nAChRs or via direct antagonist effects on 5-HT_3 _receptors. Previously, we reported that tropisetron, but not the selective 5-HT_3 _receptor antagonist ondansetron, attenuated PCP-induced cognitive deficits in mice, and that this effect of tropisetron was blocked by coadministration of the selective α7 nAChR antagonist MLA [21]. These results suggest that activation of α7 nicotinic receptors by tropisetron is likely to play a role in the mechanism of action of tropisetron [17,21]. In contrast, the selective 5-HT_3 _receptor antagonist ondansetron was reported to improve deficient auditory gating in DBA/2 mice [40]. In addition, ondansetron was shown to be effective in auditory P50 deficits, negative symptoms and cognitive symptoms in patients with schizophrenia [41-44]. These results suggest that 5-HT_3 _receptor antagonism may contribute to the action of tropisetron. Therefore, a clinical/feasibility study comparing tropisetron versus ondansetron with or without placebo will help to determine whether the addition of the α7 nAChR partial agonism of tropisetron has enhanced effects versus simply adding a 5-HT_3 _receptor antagonist. In contrast, it seems that DMXB-A addition to an atypical antipsychotic or versus placebo has the advantage of specificity of action. Nonetheless, in order to confirm the role of α7 nAChRs in the treatment of schizophrenia, a randomised double-blind, placebo-controlled study of the selective α7 nAChR agonists in patients with schizophrenia would be necessary.

There were no adverse side effects associated with the tropisetron trial. Tropisetron, which is already approved for human use outside the USA, is widely used in the treatment of patients with chemotherapy-induced or postoperative nausea and vomiting [18]. Thus, it was not surprising that tropisetron (10 mg) was well tolerated in this trial. Freedman *et al. *[14] reported that nausea occurred in 45% of a group of patients receiving a high dose of the α7 nAChR agonist DMXB-A, and this was suggested to be due to the known effects of nicotinic agonists on gastrointestinal mobility. Considering the high incidence of nausea among patients treated with α7 nAChR agonists (for example, DMXB-A), it is likely that α7 nAChR agonists (for example, tropisetron) with 5-HT_3 _receptor antagonism will be suitable therapeutic drugs for schizophrenia, since 5-HT_3 _receptor antagonists are therapeutic drugs for nausea [45].

It has been reported that varenicline, a partial agonist at α4β2 nAChRs as well as a full agonist at α7 nAChR agonists, was not effective in the auditory P50 deficits in patients with schizophrenia [46]. The precise reasons underlying the lack of varenicline on P50 deficits is currently unknown. One possibility is that the receptor desensitisation may occur by the time measurement because varenicline is a full agonist at the α7 nAChRs. A further detailed study using a selective partial agonist and a selective full agonist will be necessary. Another possibility is that there was incomplete absorption of a single dose of varenicline, leading to insufficient levels of the drug in the brain [46]. Furthermore, Freedman [47] reported the case of a patient with schizophrenia who received varenicline and experienced an activated psychotic relapse. The US Food and Drug Administration (FDA) and the Institute for Safe Medication Practices stated that serious neuropsychiatric symptoms including changes in behaviour, agitation, depressed mood, suicidal ideation and attempted and completed suicide have occurred in patients with taking varenicline [48]. Therefore, close monitoring of patients prescribed this drug will be warranted. In contrast, tropisetron and DMXB-A have not been reported to induce psychosis in patients with schizophrenia (this study, and [14]). Therefore, it is likely that tropisetron and DMXB-A have lower risk of inducing psychosis than varenicline although the reasons underlying this difference are currently unknown.

Inhibitory interneurons with α7 nAChRs are possible candidates for medication to ameliorate the habituation of auditory responses in the hippocampus, because activation of the interneurons via α7 nAChRs would increase the inhibitory synaptic input to pyramidal neurons and thereby diminish the responsiveness of these pyramidal neurons to sensory stimulation [49]. This parallels a study of postmortem human tissue that documented a decreased expression of hippocampal α7 nAChRs in schizophrenic patients [49]. Furthermore, it has been reported that [^125^I]α-bungarotoxin binding to α7 nAChRs is reduced in the thalamic reticular nucleus of schizophrenic subjects [50], and that α7 nAChR protein levels are reduced in the frontal cortex in patients with schizophrenia [51]. Thus, it seems that schizophrenic patients have fewer α7 nAChRs in the hippocampus, a condition which may lead to failure of cholinergic activation of inhibitory interneurons, clinically manifested as decreased gating of the response to sensory stimulation [17]. Therefore, it is of great interest to study whether the density of α7 nAChRs is altered in the intact brain of patients with schizophrenia. A positron emission tomography (PET) study using the selective α7 nAChR ligand [^11^C]CHIBA-1001 [52,53] in the intact brains of patients with schizophrenia is currently underway.

Finally, several limitations of this study should be mentioned. One of the main limitations of this trial was its small sample size (total n = 40) and the use of only one dose (10 mg) of tropisetron. The dose (10 mg) of tropisetron used in this study was well tolerated and resulted in significant effects on P50 deficits, and aspects of cognitive performance such as attention. However, it is currently unclear if other dosing approaches would be more efficacious. Very recently, using [^11^C]CHIBA-1001 and PET, we found that a single oral administration of tropisetron (5, 10 or 20 mg), but not ondansetron, could bind to α7 nAChRs in the intact human brain in a dose-dependent manner (Ishikawa M, Ishii K, Wu J, Toyohara J, Sakata M, Oda K, Kimura Y, Iyo M, Ishiwata K, Hashimoto K. *unpublished results*). Therefore, a randomised double-blind, placebo-controlled study of higher doses (for example, 20 mg) of tropisetron in patients with schizophrenia using a larger sample will be needed. In addition, the duration of treatment in this trial (8 weeks) was fairly short. A double-blind, placebo-controlled study with a longer duration (for example, 48 weeks) of tropisetron treatment would also be of use. Another limitation was the possible presence of practice effects for CANTAB [31], which may have been responsible for the improvement of cognitive performance in the placebo group of this study. In this study, sustained visual attention measured with the RVP subtest was significantly improved by tropisetron, but not by placebo, in non-smokers, suggesting that the effects of tropisetron on RVP might not be due to practice effects. Furthermore, Freedman *et al. *[14] also observed practice effects in a study using the MATRICS Consensus Cognitive Battery to investigate the efficacy of DMXB-A. Therefore, further detailed studies to validate the use of other cognitive batteries in patients taking tropisetron, such as MATRICS, Brief Assessment of Cognition in Schizophrenia (BACS) [54], and the CogState Schizophrenia Battery [55], would be of interest.

In conclusion, the results of the present feasibility study investigating adjunctive tropisetron as a treatment strategy for cognitive deficits in schizophrenia are promising. Similar to ondansetron and DMXB-A, tropisetron was well tolerated in this trial, and was associated with no untoward effects. If these initial pilot findings are confirmed in larger randomised controlled trials, tropisetron will be a potential therapeutic drug for the treatment of cognitive deficits and QOL in patients with schizophrenia.

## Competing interests

KH and MI have a patent for 'the use of tropisetron in neuropsychiatric diseases' through Chiba University. All authors report no competing interests related with this study.

## Authors' contributions

AS, YS, TN, THash, TY, THase, THar, NK, TS, MF, GF, MN, and MI recruited the patients enrolled in this study. AS, YS, MI and KH conducted the statistical analysis. AS and KH wrote the manuscript. KH is the principal investigator of this study. All authors read and approved the final manuscript.

## Supplementary Material

Additional file 1 **Effect of tropisetron on auditory sensory gating P50 deficits in all patients with schizophrenia**. The ratio of test P50 amplitude to conditioning amplitude was measured at baseline and 8 weeks after placebo or tropisetron treatment. Tropisetron, but not placebo, significantly decreased the P50 ratio in patients with schizophrenia. Data are the mean of the placebo group (n = 14) and the tropisetron group (n = 16). Click here for file

Additional file 2 **Supplemental table**. Scores for Positive and Negative Syndrome Scale (PANSS) in patients with schizophrenia. Click here for file

Additional file 3 **Supplemental table**. Changes of Quality of Life Scale (QLS) scores in patients with schizophrenia. Click here for file
