# Supplementary figures and images for: A randomised, double-blind, placebo-controlled trial of tropisetron in patients with schizophrenia
Source: Ann Gen Psychiatry. 2010 Jun 24;9:27. doi: 10.1186/1744-859X-9-27 (PMC2901366; doi:10.1186/1744-859X-9-27)

## Slide 1
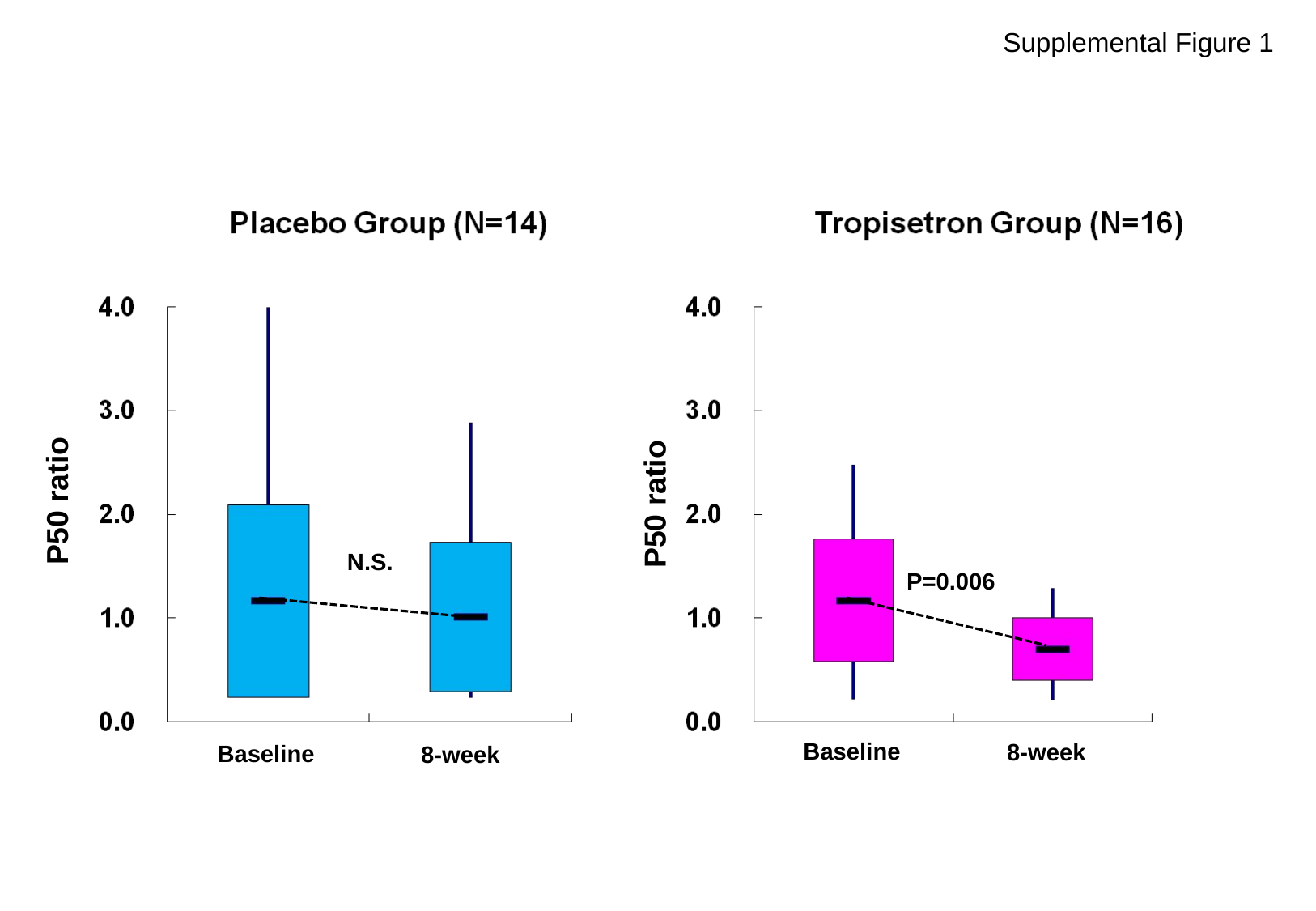

Supplemental Figure 1
P50 ratio
P50 ratio
N.S.
P=0.006
Baseline
8-week
Baseline
8-week

Supplement: Additional file 1 — Effect of tropisetron on auditory sensory gating P50 deficits in all patients with schizophrenia. The ratio of test P50 amplitude to conditioning amplitude was measured at baseline and 8 weeks after placebo or tropisetron treatment. Tropisetron, but not placebo, significantly decreased the P50 ratio in patients with schizophrenia. Data are the mean of the placebo group (n = 14) and the tropisetron group (n = 16). [file 1744-859X-9-27-S1.PPT]
