# Supplementary material for: A randomised, double-blind, placebo-controlled trial of tropisetron in patients with schizophrenia
Source: Ann Gen Psychiatry. 2010 Jun 24;9:27. doi: 10.1186/1744-859X-9-27 (PMC2901366; doi:10.1186/1744-859X-9-27)
Supplement: Additional file 2 — Supplemental table. Scores for Positive and Negative Syndrome Scale (PANSS) in patients with schizophrenia. [file 1744-859X-9-27-S2.DOC]

**Supplemental Table 1. Scores for PANSS in patients with schizophrenia**

Placebo group (n=17) Tropisetron group (n=16)

Baseline 8-week Baseline 8-week

PANSS

Total score 63.8 ± 15.0 63.0 ± 15.4 62.7 ± 16.5 63.0 ± 15.8

Positive symptoms subscale 11.9 ± 3.29 11.5 ± 3.34 11.6 ± 3.48 11.6 ± 3.38

Negative symptoms subscale 18.1 ± 6.27 17.8 ± 6.60 17.9 ± 3.48 18.0 ± 3.38

General psychopathological 33.9 ± 7.65 33.7 ± 7.68 33.1 ± 9.06 33.4 ± 8.16

symptoms subscale

PANSS: Positive and Negative Syndrome Scale

Data show the mean ± SD.
