# Supplementary material for: A randomised, double-blind, placebo-controlled trial of tropisetron in patients with schizophrenia
Source: Ann Gen Psychiatry. 2010 Jun 24;9:27. doi: 10.1186/1744-859X-9-27 (PMC2901366; doi:10.1186/1744-859X-9-27)
Supplement: Additional file 3 — Supplemental table. Changes of Quality of Life Scale (QLS) scores in patients with schizophrenia. [file 1744-859X-9-27-S3.DOC]

**Supplemental Table 2. Changes of QLS scores in patients with schizophrenia**

Placebo group Tropisetron group

Total subjects (n=33) Baseline 8-week Baseline 8-week

QLS score 78.1 ± 16.5 (n=17) 78.1 ± 17.3 (n=17) 72.6 ± 15.1 (n=16) 73.3 ± 14.7 (n=16)*

Non-smoking subjects (n=24) Baseline 8-week Baseline 8-week

QLS score 73.8 ± 15.7 (n=12) 74.8 ± 16.7 (n=12) 71.6 ± 15.8 (n=12) 72.0 ± 15.4 (n=12)

Data show the mean ± SD.

*P=0.029 (vs. baseline).

QLS: Quality of Life Scale
